# Case report: Ruxolitinib plus dexamethasone as first-line therapy in haemophagocytic lymphohistiocytosis

**DOI:** 10.3389/fonc.2023.1054175

**Published:** 2023-03-02

**Authors:** Lin Zhao, Hui Yang, Wei-ying Qu, Ying-jia Lu, Zhou Feng

**Affiliations:** ^1^ Department of Haematology, Shuguang Hospital Affiliated to Shanghai University of Traditional Chinese Medicine, Shanghai, China; ^2^ Department of Clinical Laboratory, Shuguang Hospital Affiliated to Shanghai University of Traditional Chinese Medicine, Shanghai, China

**Keywords:** haemophagocytic lymphohistiocytosis, ruxolitinib, dexamethasone, first-line, therapy

## Abstract

Haemophagocytic lymphohistiocytosis (HLH) is a cytokine-driven inflammatory syndrome caused by uncontrolled hypersecretion of inflammatory cytokines. Conventional first-line treatment for HLH included HLH-94 and HLH-2004 regimens. However, quite a few patients do not respond to treatment or cannot tolerate intensive chemotherapy. We reported two cases of HLH, one caused by natural killer (NK)/T-cell lymphoma and another associated with missense variants in the *perforin 1* gene. They both received the ruxolitinib plus dexamethasone protocol and had a rapid response to treatment without obvious adverse effects. Our report indicates that treatment with ruxolitinib plus dexamethasone might be a potential option for HLH, and clinical trials warrant further investigation. In addition, the detection of HLH-related genes is necessary for the identification of late-onset familial HLH in certain settings.

## Introduction

Haemophagocytic lymphohistiocytosis (HLH) is a rare, life-threatening, and rapidly progressive syndrome characterized by hyperinflammation caused by inherited or acquired immune dysregulation. Primary HLH often refers to patients with clear familial inheritance or genetic causes. Autosomal recessive inheritance is a common mode of inheritance of this disease [also known as familial hemophagocytic lymphohistiocytosis (FHL)]. Secondary HLH (sHLH) is typically triggered by malignant, infectious, or autoimmune/autoinflammatory stimuli, often without known HLH pathogenic genetic disorders and family history (1). Due to its rapid progression and high mortality, timely initiation of appropriate treatment is critical to improving prognosis.

The treatment of HLH is generally divided into two stages. First, induction regimens mainly target the excessive inflammatory state to control the progression of HLH. Then, aetiologic therapy focuses on correcting the underlying immune deficiency and controlling the primary disease to prevent HLH recurrence. The etoposide-based HLH-94 and HLH-2004 regimens remain widely accepted as the standard treatment for HLH. Nevertheless, quite a few patients do not respond to treatment or are unable to tolerate intensive chemotherapy (2, 3). The Janus kinase (JAK) 1/2 inhibitor ruxolitinib (RUX) is a promising option for the treatment of HLH (4–6), and combined glucocorticoid therapy might further improve the efficacy. As of September 2022, eighteen human studies have been reported evaluating RUX in patients with HLH. Sixteen of these studies used RUX in sHLH as a first-line or salvage setting, with a dozen case reports each consisting of a single patient. However, reports using RUX as a first-line therapy among patients with active malignancy or primary HLH are scarce (7). Herein, we report 2 cases of HLH (1 case of sHLH associated with natural killer (NK)/T-cell lymphoma, and 1 case of primary HLH with missense variants in the *perforin gene* (*PRF1*), highlighting the contribution of RUX plus dexamethasone (DXM) in controlling hyperinflammation in sHLH or primary HLH.

## Case presentation

### Case 1

**Figure 1:**
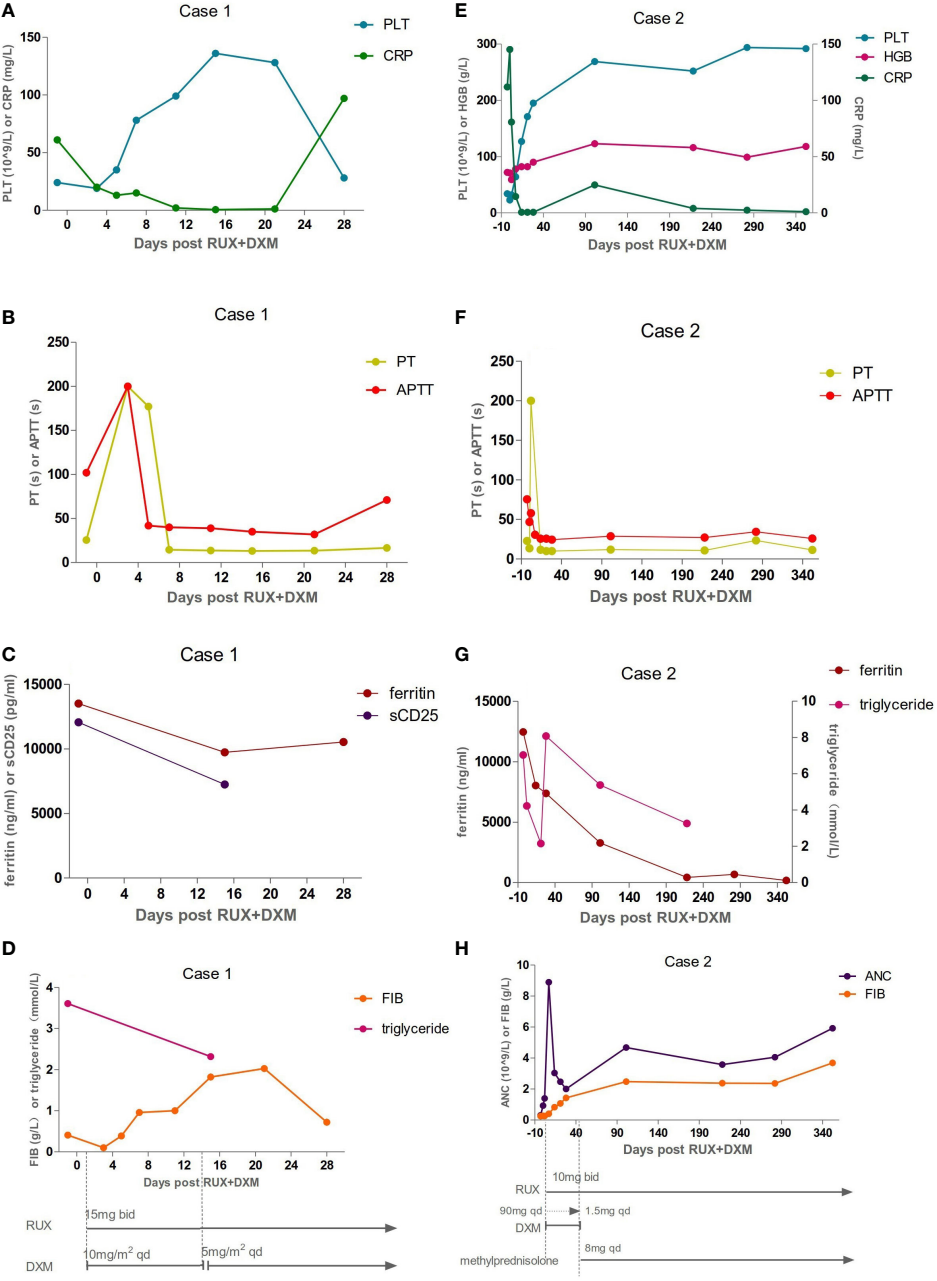
Inflammatory, coagulation function, and hematologic responses to HLH therapies: Day 0 corresponds to ruxolitinib (RUX) initiation. Treatment schedules, lines, and bars represent continuous therapy and individual doses of drugs with intermittent schedules. Arrows indicate that treatment was continued. **(A–D)** The laboratory assessments of Case 1. The levels of platelet (PLT), prothrombin time (PT), active thromboplastin time (APTT), C-reactive protein (CRP), fibrinogen (FIB), ferritin, soluble CD25 (sCD25), and triglyceride were abnormal before Day 0 and gradually improved after therapy. But he developed a fever again on Day 28. The levels of ferritin and CRP were all elevated. The values of FIB and PLT were reduced again. **(E–H)** The laboratory assessments of Case 2. The dexamethasone (DXM) dose was reduced by half every three days, finally reaching a reduced dose of 1.5 mg on Day 42. It was replaced by methylprednisolone on Day 43. Inflammatory, coagulation function, and hematologic parameters were abnormal before Day 0, and gradually improved after therapy.

A previously healthy 27-year-old man presented with skin ulceration and exudation in January 2022. He received antibiotics. Three months later, fever, generalized body swelling, and fatigue were experienced by the patient. The laboratory data revealed abnormal liver functions, with an alanine aminotransferase (ALT) level of 92 units/L (normal: 7-40 units/L), aspartate aminotransferase (AST) level of 181.3 units/L (normal: 13-35 units/L), and total bilirubin (TBil) level of 73 µmol/L (normal: 0-23 µmol/L). The laboratory data also revealed thrombocytopenia with a platelet (PLT) count of 24 × 10^9^/L (**Figure 1A**). The level of haemoglobin (HGB) was 74 g/L. His ferritin level was 13504 ng/ml (normal: 23.9-336.2 ng/ml), and triglyceride (TG) level was 3.61 mmol/L (normal: 0-1.7 mmol/L) (**Figure 1C, D**). He had severe hypoalbuminemia (20 g/L). Epstein-Barr virus (EBV)-DNA in blood plasma was 3.46 × 10^3^ copies/ml (normal: < 400 copies/ml). The level of soluble CD25 (sCD25) was 12062 pg/ml (normal: ≤ 6000 pg/ml) (**Figure 1C**). The patient tested negative for human immunodeficiency virus (HIV). He denied a personal and family history of haematological pathologies, specifically HLH.

The patient’s symptoms worsened, as evidenced by deteriorated coagulopathy with a prothrombin time (PT) of 25.5 s (normal: 10.4-12.7 s) and an active thromboplastin time (APTT) of 102 s (normal: 22.3-32.5 s) (**Figure 1B**). The fibrinogen level was only 0.41 g/L (normal: 1.8-3.5 g/L) (**Figure 1D**). Bone marrow aspiration and biopsy demonstrated inflammatory changes and an increase in the number of macrophages and histiocytes with intense haemophagocytosis. Therefore, the patient fulfilled 7 of 8 diagnostic criteria for HLH (**Table 1**). NK-cell activity was not analysed due to the lack of availability in Shanghai during the pandemic.

**Table 1:** Clinical and laboratory parameters measured in both patients at diagnosis and Day 14 post-ruxolitinib (post-RUX).

Parameter	Proposed HLH diagnostic criteria (requires 5/8) (8)	Data of Case 1		Data of Case 1		
		at diagnosis	Day 14 post-RUX	at diagnosis	Day 14 post-RUX	
Fever	≥38.5°	Present	No	No	No
Spleen	Splenomegaly	Present	No	Present	No
Bicytopenia	HGB <90 g/L, and/or PLT <100× 10^9^/L, and/or ANC< 1.0× 10^9^/L	74 g/L 24 × 10^9^/L 2.1× 10^9^/L	87 g/L 136 × 10^9^/L 3.6× 10^9^/L	93 g/L 37 × 10^9^/L 0.3 × 10^9^/L	82 g/L 127 × 10^9^/L 3.04 × 10^9^/L
Hypertriglyceridemia and/or hypofibrinogenemia	>3 mmol/L < 1.5 g/L	3.61 mmol/L 0.41 g/L	2.32 mmol/L 1.82 g/L	7.04 mmol/L 0.25 g/L	4.23 mmol/L 0.83 g/L
Hyperferritinemia	≥500 μg/L	13504 μg/L	9728 μg/L	12459 μg/L	8034 μg/L
Soluble CD25	Elevated	Yes (12062 pg/ml)	Yes (7238 pg/ml)	Not tested	Not tested
NK Cell Activity	Absent or Low	Not tested	Not tested	Not tested	Not tested
Haemophagocytosis	Observed in BM, LN, spleen, or liver	Observed in BM	Not valued	Observed in BM	Not observed in BM
Alternatively:					
Genetics	Homozygosity or compound heterozygosity for HLH-associated mutations in an appropriate clinical setting	Not tested		*PRF1* and *LYST*		

HLH, Haemophagocytic lymphohistiocytosis; HGB, Haemoglobin; PLT, Platelet; ANC, Absolute neutrophil count; BM, Bone marrow; LN, Lymph node; *PRF1*, The perforin gene; *LYST*, Lysosomal trafficking regulator.

The patient experienced extreme fatigue, severe oedema, persistent high fever, and large skin ulcerations with exudation. He became expressionless and unresponsive. We decided to start with RUX 15 mg twice daily plus dexamethasone (DXM) 10 mg/m^2^/day per HLH-94 dosing, not only because of his poor performance status (PS) and progressive deterioration of liver function but also because of the short supply of blood products, including red blood cells and platelets, at specific periods in Shanghai. Fourteen days later, DXM was reduced to 5 mg/m^2^/day. Broad-spectrum antibiotics, fibrinogen, prothrombin complex, and immunoglobulin were also given. The patient had no fever on Day 3 (Day 0 corresponds to RUX initiation). Eighty percent of skin ulcers were healed on Day 7. Oedema and fatigue symptoms were significantly relieved. The TBil level dropped to 58 µmol/L. After two weeks of therapy, the PLT count recovered to 136 × 10^9^/L (**Figure 1A**). The fibrinogen level, PT, and APTT improved (**Figure 1B, D**). The sCD25 level decreased to 7238 pg/ml (**Figure 1C**). The patient achieved a partial response (PR) according to the response criterion described in previous studies (9, 10). He underwent two skin biopsies and a cervical lymph node biopsy by a core needle, but all were negative for lymphoma. The histopathology of the excised right inguinal lymph node indicated an NK/T-cell lymphoma on Day 22. The pegaspargase + Gemox regimen was started on Day 23. He developed a fever again on Day 28. The levels of C-reactive protein (CRP, 97 mg/L) and ferritin (10532 ng/ml) were all elevated (**Figure 1A, C**). The values of PLT (31 × 10^9^/L) and fibrinogen (0.80 g/L) were reduced again (**Figure 1A, D**). The pulmonary computed tomography (CT) scan suggested pneumonia. The result of alveolar lavage fluid culture was *Pseudomonas aeruginosa.* Although broad-spectrum antibiotics were administered according to the drug susceptibility, the patient died on Day 33 with incomplete induction chemotherapy.

### Case 2

A 33-year-old female with a history of encephalomyelitis treated with high-dose methylprednisolone in 2018 presented with neutropenia and mild anaemia in November 2021. A month later, she developed a high fever, fatigue, headache, and pancytopenia with an absolute neutrophil count (ANC) of 0.3 × 10^9^/L, HGB of 93 g/L, and PLT of 37 × 10^9^/L (**Figure 1E, H**). The CRP level was 131 mg/L (normal: 0-8 mg/L) (**Figure 1E**). She was admitted to our hospital. Bone marrow aspiration and biopsy demonstrated haemophagocytosis. Flow cytometric (FC) analysis of bone marrow showed no clonal abnormalities, and chromosome karyotype analysis was normal. The laboratory data revealed abnormal liver function with an ALT of 70 units/L, AST of 182 units/L, and TBil of 24 µmol/L. Coagulation detection showed a decreased level of fibrinogen (0.25 g/L) and prolonged APTT (75.4 s) (**Figure 1F, H**). Her ferritin level was 12459 µg/L and the TG level (7.04 mmol/L) was four times higher than normal (**Figure 1G**). EBV-DNA in blood plasma was 896.68 copies/ml (normal: <400 copies/ml). EBV nuclear antigen IgG (EBNA-IgG) and EBV capsid antigen IgG (EBCA-IgG) antibody titers were 235 U/ml (normal: < 5 U/ml) and 218 U/ml (normal: < 20 U/ml), respectively. Abdominal CT showed splenomegaly. The patient denied that she had repeatedly developed HLH and denied a family history of HLH. According to her clinical manifestations and laboratory parameters, she was diagnosed with HLH (**Table 1**).

Magnetic resonance imaging (MRI) showed multiple inflammatory demyelinating lesions in the bilateral paraventricular area, bilateral radiation crown area, and bilateral frontoparietal temporal cortex. Cerebrospinal fluid pressure was high, protein content was normal, and no tumour cell infiltrations were found. The recurrence of encephalomyelitis was considered. No clear malignant, autoimmune, or infectious triggers for HLH were identified. EBV infection was suspected as a potential trigger for her HLH. The level of sCD25 and NK-cell activity were unavailable during this time in Shanghai.

The patient was given 90 mg DXM a day for 3 days. At the same time, RUX 10 mg was administered orally twice a day. The DXM dose was reduced by half every three days, finally reaching a reduced dose of 10 mg. After that, the dose was adjusted every two weeks to 1.5 mg according to the patient’s laboratory examination results. Then, DXM was replaced with 8 mg methylprednisolone for maintenance treatment. Her fever was controlled on Day 2. The PLT and ANC counts had normalized on Day 7 (**Figure 1E, H**). The ferritin, fibrinogen, and TG level improved on Day 14 (**Figure 1G, H**). She achieved PR according to the response criterion on Day 14 (9, 10).

**Figure 2:**
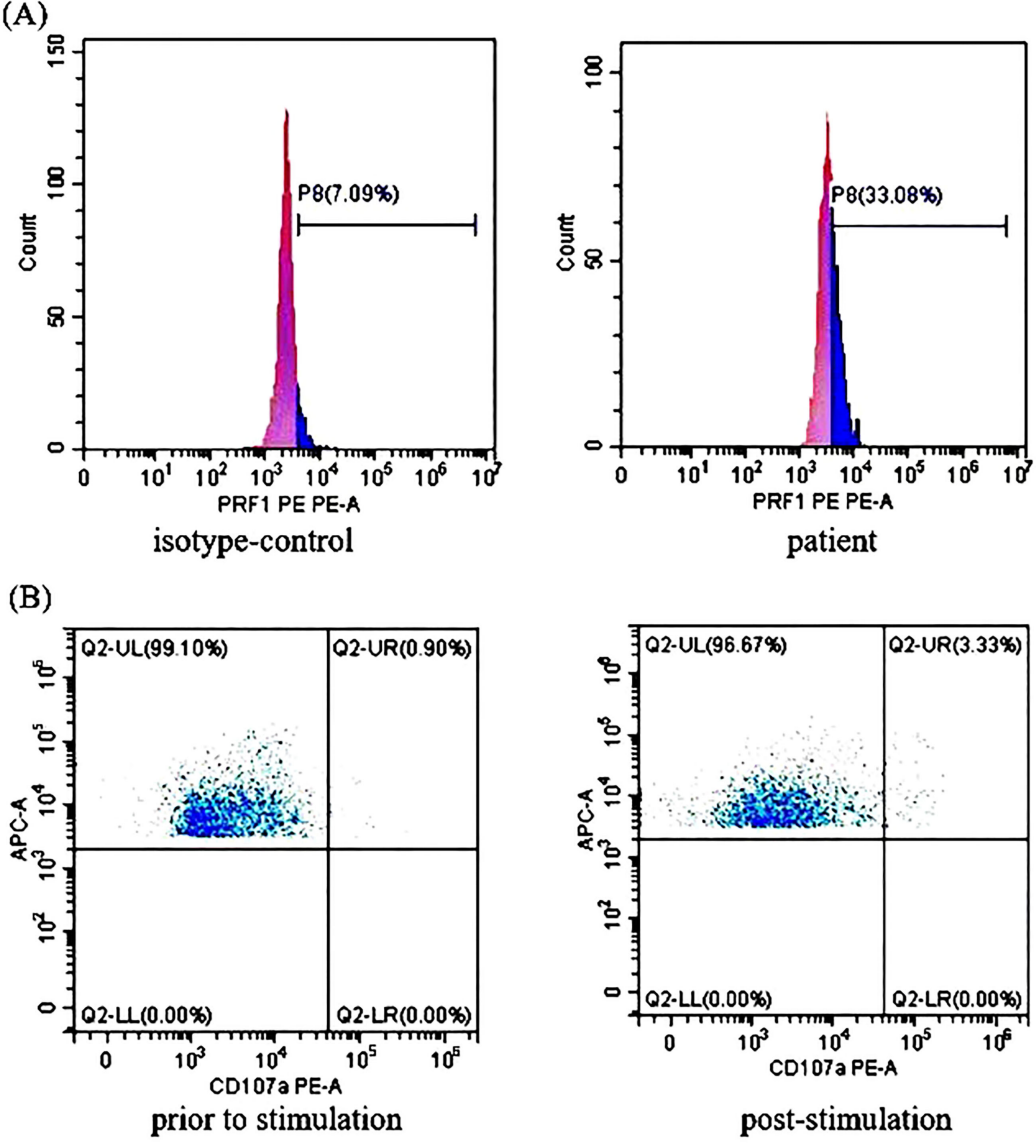
The perforin protein (PRF1) expression and ΔCD107a in natural killer (NK) cells of Case 2: **(A)** Flow cytometry analysis revealed the decreased expression rates of PRF1 in NK cells of Case 2. The expression rates of PRF1 were 7.09% of the negative control and 33.08% of the patient sample. The negative control was treated with isotype-control antibodies to eliminate the false positive interference caused by the non-specific binding of antibodies to the cells. Thus, the actual expression rates of PRF1 were 25.99% (i.e., 33.08% - 7.09% = 25.99%, normal: ≥81%). **(B)** The NK cells stimulation test showed severe dysregulation of the immune response. The expression rates of CD107a in the NK cells of Case 2 were 0.90% (before stimulation) and 3.33% (post-stimulation). Thus, ΔCD107a was 2.43% (ΔCD107a >10% indicates normal degranulation function).

Treatment with RUX and a low dose of methylprednisolone were continuous. Plasma EBV-DNA load was 423 copies/ml on Day 265. EBNA-IgG and EBCA-IgG antibody titers were 185 U/ml and > 750 U/ml on Day 273, respectively. Two antibodies were still positive with high avidity. The onset of primary HLH in adults may lead clinicians to ignore or even misdiagnose the disease. To further clarify the aetiology of HLH in Case 2, the next-generation sequencing (NGS) of HLH-related genes was performed on Day 275. A mutation of the *PRF1* gene, c.1349C > T (p. T450M) was reported. Two mutations of the lysosomal trafficking regulator (*LYST*) were also detected: c.1183C > T (p. R395C) and c.2183G > T (p. S938I). All of them were heterozygous missense mutations. The NGS results are shown in **Table 2**. Unlike *PRF1* mutations, which are known pathogenic variants responsible for FHL, the clinical significance of the two *LYST* gene variants is unclear. FC analysis revealed the decreased expression of PRF1 in NK cells (**Figure 2A**). NK cells stimulation test showed severe dysregulation of immune response (**Figure 2B**) on Day 352. The final diagnosis of the patient was FHL type 2 from homozygous c.1349C > T (p. T450M) missense variants in the *PRF1* gene. The patient’s parents were reluctant to test HLH-related genes for financial stress, so it was impossible to determine the location of these variants. She remained well without neurological symptoms at the monthly follow-up. The brain MRI on Day 329 showed stable disease without any new lesions. The ferritin level, liver function, coagulation, and hematologic parameters were normal at the last follow-up on Day 353 (**Figure 1E, F, G, H**). The patient is currently enjoying excellent PS.

**Table 2:** Genetic test* results of Case 2.

	Chromosome position	Transcript mutation position	Nucleotide change	Amino acid change	Homozygous/heterozygous	Disease/ phenotype	Genetic mode
*PRF1*	chr10: 72358128	NM_001083116 Exon3	c.1349C >T	p. T450M	Heterozygous	FHL2	AR
*LYST*	chr1: 235972935	NM_00008.4 Exon5	c.1183C > T	p. R395C	Heterozygous	CHS	AR
*LYST*	chr1: 235969623	NM_00008.4 Exon6	c.2813G >T	p. S938I	Heterozygous	CHS	AR

*PRF1*, The perforin gene; *LYST*, Lysosomal trafficking regulator; AR, Autosomal recessive inheritance; FHL2, Familial haemophagocytic lymphohistiocytosis 2; CHS, Chediak‑Higashi.

* HLH-related genes were detected using high-throughput sequencing (sequencing type, targeted sequencing; average sequencing depth, 115X; mutation types, SNV, Indel, CNV).

## Discussion

HLH-94 and HLH-2004 are the recommended first-line treatment protocols for HLH, but they are all based on clinical studies of children with HLH. There is no consensus on HLH in adults, especially for patients with severe cytopenia or poor PS. Less toxic treatment for HLH has emerged as a subject of intense interest and study (11).

RUX, an inhibitor of JAK 1 and JAK 2, blocks cytokine signaling through JAK/STAT and inhibits the action of proinflammatory cytokines. In murine models, treatment with RUX significantly lessened the clinical and laboratory manifestations of primary HLH and sHLH (12). Since Broglie L et al. first reported the use of RUX in a refractory HLH paediatric case of unknown aetiology (13), there has been an increasing number of publications describing the use of this drug in patients with HLH. Zhang et al. presented the largest prospective study reported to date, enrolling fifty-two newly diagnosed paediatric patients, demonstrating the clinical benefit of RUX as a first-line targeted therapy. RUX has a rapid efficacy for paediatric HLH with few attributable serious adverse events (6).

For HLH secondary to aggressive lymphoma, controlling HLH with fewer side effects as soon as possible could create conditions for lymphoma chemotherapy (14). Although the outcome of Case 1 was disappointing, HLH was controlled and the induction therapy was initiated. The cause of failure may be rapid tumour progression or infection caused by intensive chemotherapy and/or RUX plus DXM. Therefore, for highly suspected aggressive lymphoma, it is important to clarify the diagnosis and initiate aetiologic treatment as soon as possible.

Allogeneic haematopoietic stem cell transplantation (allo-HSCT) is the only available curative treatment for FHL at present. The success of HSCT is dependent on complete control of the disease prior to transplantation (15). Case 2 describes a patient with FHL successfully treated in first intention by a combination of RUX and DXM. Our observation suggests that this less toxic and effective treatment regimen could be used as a first-line therapy for FHL and help bridge eligible patients to HSCT even if Case 2 abandoned HSCT for personal reasons.

The management of HLH often requires adjustment of guideline-directed therapies due to treatment-related toxicity or the complexities of managing the underlying trigger and resultant hyperinflammation simultaneously. Our two cases had poor PS and could not tolerate the standard protocol containing chemotherapy. In this setting, the application of RUX was a good option. Both patients had a prompt response to RUX plus DXM. The initial manifestation of the treatment response was rapid control of fever, followed by improvement of abnormal coagulation and PLT counts. Consistent with other reports, they both appeared to be well-tolerated (16, 17).

RUX combined with glucocorticoids or conventional chemotherapy may further improve efficacy. However, there is no conclusion as to which combination therapy is better. RUX plus glucocorticoids are frequently used. There is no definitive conclusion on whether glucocorticoids, methylprednisolone, or DXM should be used when initiating RUX therapy. In addition, the optimal dose and schedule of glucocorticoid administration remain to be determined. A minimum of 10 cases were arbitrarily chosen to identify larger studies. At the time of writing this report, 3 independent larger studies describing 122 unique patients have been published. In one retrospective study, 36 patients with lymphoma-associated haemophagocytic syndrome were treated with RUX combined with doxorubicin, etoposide, and dexamethasone (DEP) (18). In two single-centre prospective studies, 34 refractory/relapsed HLH patients and 52 paediatric HLH patients were enrolled (5, 6). According to the protocols of these prospective studies, glucocorticoids could be continued if the patient was receiving them before enrolment. In Case 1, we initiated treatment with RUX at a dose of 15 mg twice daily. Because the cause of HLH was not clear, DXM 10 mg/m^2^/day per HLH-94 dosing was used. In Case 2, we used high-dose DXM because the patient’s presentation was complicated with recurrent encephalomyelitis. Similar to HLH, glucocorticoids have been the mainstay of treatment in encephalomyelitis.

In both cases, abnormal elevation of EBV-DNA was detected. During the follow-up of Case 2, persistent slight abnormalities of serum EBV-DNA were revealed even though the patient’s clinical symptoms improved significantly. EBV infection may play an important role in the occurrence of haemophagocytosis in Case 2. EBV may be involved in malignancy-related HLH, rheumatic immune disease-related HLH, or primary HLH with known genetic defects. For somewhat unclear reasons, EBV is highly associated with HLH in Asia. In one report, it was observed to be associated with nearly 3/4 of HLH patients (19). Sustained primary EBV infection can trigger this immediately fatal disorder, especially in patients with unknown congenital or acquired immunodeficiencies (20, 21). In addition, EBV-DNA copies in whole blood and plasma of EBV-HLH patients before and after RUX treatment did not change, indicating that RUX improves inflammation without affecting the underlying primary cause of HLH (5).

Malignancy, particularly lymphoma-associated HLH, was a prominent adverse prognostic marker correlating with poorer survival in several studies (22–24). Patients with T-cell lymphoma generally had worse outcomes than those with B-cell lymphoma (25). Case 1 died despite prompt treatment for the primary disease after diagnosis.

There is an increasingly recognized overlap between primary HLH and sHLH as new cases are described and genetic discoveries are better understood. At present, it is believed that sHLH also has a genetic background, such as heterozygous changes and polymorphisms of primary HLH-related genes, and exhibits HLH pathogenesis after suffering the “second hit” of external trigger factors (such as a virus infection). sHLH is much more commonly described than primary HLH in adults; therefore, adults are rarely tested for genetic abnormalities, which leads to the misdiagnosis of late-onset cases. Gene sequencing is recommended for patients with HLH whose aetiology is unknown and/or who have recurrent episodes to identify the rare late-onset case of primary HLH. Patients who have not been detected the currently known pathogenic genes of HLH and cannot be determined the secondary aetiology needs to be continuously sought in subsequent treatment and follow-up.

In Case 2, NGS revealed c.1349C > T (p. T450M) heterozygous missense variations in the coding sequence of exon 3 of the *PRF1* gene. The oldest individual ever documented to be diagnosed with FHL Type 2 from homozygous c.1349C > T (p. T450M) missense variants in the *PRF1* gene was a 33-year-old Indian man of a similar age to Case 2 (26). Perforin is encoded by the *PRF1* gene and forms pores in target cell membranes in a process highly dependent upon its C2 domain, allowing granzyme to enter and initiate caspase-mediated apoptosis of the target cell (27). Functional studies showed that the mutant perforin c.1349C > T (p. T450M) heterozygous missense variant was completely inactivated at 37°C. The scale-invariant feature transform (SIFT) predicted value was - 4.921, which was considered a harmful variant. According to the 2017 publication of the American College of Medical Genetics (ACMG) and Genomics guidelines (28, 29), it is inferred that the c.1349C > T mutation is a pathogenic mutation. The delayed onset of FHL Type 2 may be related to the mutation type (30), triggering factors (31), and pathogenic variation pattern of *PRF1* (32). A possible explanation for the delayed onset of HLH is that these patients carry heterozygous missense mutations, which encode some *PRF1* activities. Two missense mutations of *LYST* genes were also found in Case 2. The relevant literature on the mutation was not reported in the Human Gene Mutation Database (HGMD) database. According to the ACMG guidelines, the clinical significance of this mutation is uncertain.

In conclusion, RUX is effective and less toxic in both primary HLH and sHLH. However, there are still several additional questions of RUX that warrant further study, including dosing, duration, and combinations. Moreover, genetic screening is recommended to exclude adult-onset FHL to reduce patient mortality, especially for patients with unexplained recurrence of disease.

## Data availability statement

The original contributions presented in the study are included in the article/supplementary material. Further inquiries can be directed to the corresponding author.

## Ethics statement

Ethical review and approval was not required for the study on human participants in accordance with the local legislation and institutional requirements. The patients/participants provided their written informed consent to participate in this study.

## Author contributions

LZ gathered the clinical information and drafted the manuscript. LZ and HY approved the final diagnosis and formulated the therapeutic strategies. W-YQ, Y-JL, and ZF reviewed multiple drafts of the manuscript. W-YQ made the chart and picture. All authors listed have made a substantial, direct, and intellectual contribution to the work and approved it for publication.
